# Perception of Tourists Regarding the Smoke-Free Policy at Suvarnabhumi International Airport, Bangkok, Thailand

**DOI:** 10.3390/ijerph10094012

**Published:** 2013-08-30

**Authors:** Nithat Sirichotiratana, Subash Yogi, Chardsumon Prutipinyo

**Affiliations:** 1 Health Administration Department, Faculty of Public Health, Mahidol University, 420/1 Rajvithi Road, Rajthevi District, Bangkok 10400, Thailand; E-Mail: nithats@gmail.com; 2 Saath-Saath Project, FHI360, GPO Box 8803, Gopal Bhawan, Anamika Galli Baluwatar, Kathmandu, Nepal; E-Mail: subash.yogi@gmail.com; 3 Health Administration Department, Faculty of Public Health, Mahidol University, 420/1 Rajvithi Road, Rajthevi District, Bangkok 10400, Thailand

**Keywords:** tourists, smoke-free policy, smoke-free airport, perception, Thailand

## Abstract

This study was conducted during February-March 2012 to determine the perception and support regarding smoke-free policy among tourists at Suvarnabhumi International Airport, Bangkok, Thailand. In this cross-sectional study, 200 tourists (*n* = 200) were enrolled by convenience sampling and interviewed by structured questionnaire. Descriptive statistics, chi-square, and multinomial logistic regression were adopted in the study. Results revealed that half (50%) of the tourists were current smokers and 55% had visited Thailand twice or more. Three quarter (76%) of tourists indicated that they would visit Thailand again even if it had a 100% smoke-free regulation. Almost all (99%) of the tourists had supported for the smoke-free policy (partial ban and total ban), and current smokers had higher percentage of support than non-smokers. Two factors, current smoking status and knowledge level, were significantly associated with perception level. After analysis with Multinomial Logistic Regression, it was found that perception, country group, and presence of designated smoking room (DSR) were associated with smoke-free policy. Recommendation is that, at institution level effective monitoring system is needed at the airport. At policy level, the recommendation is that effective comprehensive policy needed to be emphasized to ensure smoke-free airport environment.

## 1. Introduction

Tobacco continues to be the leading global cause of preventable deaths. It is classified as a “known human carcinogen” (cancer-causing agent) by the US Environmental Protection Agency (EPA). Second hand smoke (SHS) also called environmental tobacco smoke, involuntary smoke, and passive smoke, is the combination of “sidestream” and “mainstream” smoke. It is also known to be a human carcinogen [1]. Around the world, an estimated 33% male and 35% female non-smokers are regularly exposed to SHS [2] at home and workplaces. Studies have clearly shown nonsmokers risk of developing lung cancer by 20–30% at work place or at home from exposure to SHS [3].

In 2007 smoke-free policy guidelines under article 8 of World Health Organization Framework Convention on Tobacco Control (WHO FCTC) were formed and were unanimously agreed as the safest policy against SHS [4]. Finally the convention concluded that by going 100% smoke-free and not permitting enclosed or substantially enclosed workplaces, public places, public transport, international airports and bars and restaurants can protect the health of non-smokers and smokers [5].

It has been seen that low and middle-income countries are effectively implementing smoke-free legislation, such as Kenya, Niger, Panama and Thailand [6]. Asian and developing countries harbor 70% of the world’s 1.1 billion smokers [7]. In China seven of 10 non-smoker adults are exposed to SHS [8], while Indonesia has not yet implemented the smoke-free policy as outlined by the FCTC article 8 [9]. Protecting people from SHS particularly in the indoor places such as airports, bars, and restaurants is a pivotal issue.

Some Southeast Asian countries have strong laws that offer a high standard of protection for people. However these laws do not comply with the maximum standards set forth by the FCTC since they allow Designated Smoking Rooms (DSRs). Thailand and Singapore, being the tobacco control leaders in the region, are still unable to eliminate DSRs.

A Hong Kong technical feasibility study of smoking concluded that even the best designed designated smoking rooms do not fully protect non-smokers from secondhand smoke. Some leakage of secondhand smoke is inevitable. The study also found that smoking rooms were not practical due to the technical demands and costs associated with the building, operation and maintenance of the rooms [10]. In Santiago, Chile, one study found that smoke from designated smoking rooms leaked into non-smoking areas. Non-smoking areas of venues that allowed smoking in ventilated designated smoking rooms had nicotine concentration in the air 3.2 times higher than completely smoke-free venues [11]. Recent studies document that toxins from tobacco smoke remain even after the cigarette has been extinguished; this is known as ‘third-hand’ smoke. As a result, indoor spaces become contaminated with tobacco toxins even after the visible smoke disappears [12].

A World Bank report on the global tobacco epidemic concluded that smoking restrictions can reduce overall tobacco consumption by 4–10% [13]. The United States Occupational Health and Safety Administration reported that the National Energy Management Institute estimated that clean air increases productivity by 3.5 % or 15 min per day for employees, while 94 state government office buildings surveyed indicated that poor indoor air quality attributed to 14 min per day productivity loss or 3 % [14]. Comprehensive smoke-free laws, or total ban on indoor smoking, will fully protect non-smokers from secondhand smoke exposure, and the only effective way to ensure that exposure will not occur [3].

Designated smoking rooms at the airports are to facilitate the passenger and minimize the harms from SHS but studies at airport with well-functioning designated smoking rooms in United States found evidence of secondhand smoke leakage to the indoor smoke-free areas due to the opening and closing of doors [13]. Another study in U.S done in airports as well have shown that millions of passengers and thousands of airport workers are still being needlessly exposed to serious health hazards because of the ongoing indoor smoking in a handful of major hub airports [15].

The airports are allowing ventilation systems which do not eliminate the hazard of second-hand smoke. Studies have shown that nicotine concentrations adjacent to outdoor smoking areas at airports can be as high as those in some smokers’ homes [16]. The study carried out at the airport shows that airport smoking rooms expose non-smokers in adjacent non-smoking areas to a significant concentration of nicotine vapor from SHS [17].

Thus, by adopting 100% smoke-free indoor air policies, airports can choose to save money, space, and most importantly, people’s lives—both airport employees and passengers/travelers. Gap in the law means patrons and workers still have smoke blown in their faces at workplaces and public places. These unequal protections from exposure to SHS leaves everyone at the risk of life threatening public health effects relate to heart attack, cancer and direct health costs [18]. Thus, this study, by finding out the knowledge and perception of the tourists on smoke-free policy at the airport along with their support for the 100% smoke-free law, would be helpful in advocating the airport for total smoking ban; ultimately saving lives rather than killing people by allowing ventilation rooms inside the airport.

## 2. Experimental Section

### 2.1. Research Design

A cross-sectional study was conducted to explore the knowledge, perception and support on smoke-free airport policy among the tourists at Suvarnabhumi International Airport, about 25 km east of Bangkok. In the study quantitative methods and tools were used to gather the information in order to quantify the variables under the study.

The target population for the study were the tourist passengers, *i.e.*, non-Thai, above 18 years of age. Since the number of tourists passing through the airport was dynamic, the sample size depended on the season and time of the day. Therefore, researchers employed an incidental (purposive) sampling method among departing tourists to be included in this pilot study. For calculating the sample size in this study was conducted in order to estimate the prevalence of perception on smoke-free policy which was found to be 0.86. Thus at 95% CI with 5% level of significance sample size of 180 was determined for the study.

Also another method was used to get to the conclusion. By projecting a high rejection rates during the interview process, an additional 20 samples had been taken as precaution for error from data collection. Thus, 200 incidental samples were collected for the study.

### 2.2. Research Instruments

A structured questionnaire were used to obtain the information on knowledge, perception and support on smoke-free policy from the tourists through interview. The questionnaire was constructed based on a social science research method with measurement on a Likert scale with 5 levels: “Strongly Disagree”, “Somewhat Disagree”, “Neutral”, “Somewhat Agree”, and “Strongly Agree”. “Strongly Disagree” equals to 1, while “Strongly Agree” equals to 5. The structured questionnaire had closed ended, multiple response and multiple choice questions. The questionnaires were divided into three sections. The questionnaire was pre-tested among 30 international students for clarity, validity and reliability. Furthermore, questions were reviewed by tobacco control literature and experts. Finally, consensus on the tools for measuring the research objectives was reached by close consultation with experts. For measuring the perception, all 8 items from questionnaire were computed as a whole. After the scales of these eight items were computed, we tested for reliability. Cronbach’s alpha coefficient of 0.81 was obtained.

### 2.3. Data Collection

Prior to the data collection, permission from the related authorities were obtained and ensured. The researcher along with an assistant collected the data at the airport from the tourists. During the data collection researcher first provided the information sheets and explained the objectives and procedure of data collection to the respondents, and obtained verbal and written informed consent voluntarily prior to the interview. The researchers received approval from Ethical Review Committee for Human Research, Faculty of Public Health, Mahidol University, certificate of approval number MUPH 2012-031.

### 2.4. Data Analysis

For data analysis, computerized statistical software for data analysis were used. Descriptive statistics included frequency, percentage, mean and standard deviation. These were applied for describing characteristics of samples, knowledge and perception levels. Data were presented in terms of percentage, mean and standard deviation as per descriptive analysis. Chi-Square, Multinomial Logistic Regression analysis were used for testing association with support for the 100% smoke-free policy.

## 3. Results and Discussion

### 3.1. Findings from Descriptive Statistics

#### 3.1.1. General Characteristics of the Respondents

Table 1 below describes the general characteristics of the 200 respondents. The distribution of the general characteristics like age, sex, education, marital status and smoking status are mentioned in the table. The table also provides information on the number of visits made to Thailand by the tourists and the geographical region they belong to, *i.e.*, residence of the respondents. In addition, their views on coming to Thailand when it goes 100% smoke-free were also described in the table.

Exploration at the smoking status of the respondents, surprisingly revealed that exactly half of the respondents taken in the study were currently smokers where as 40% had never smoked and 10% had given up smoking. Similarly, it was also found that 45.1% respondents were on their first visit to Thailand and 29% had visited over three times. Likewise on analyzing the respondents’ opinion on coming to Thailand when it goes 100% smoke-free; two-third (65.8%) of the respondents replied that they would like to come to Thailand, whereas 12% were of the opposite view and the rest were unsure.

**Table 1:** General Characteristics of Respondents.

Variables	Number	Percent
**Age Group/years (*n* = 199)**		
18–35	114	57.0
35+	86	43.0
**Sex (*n* = 200)**		
Male	126	63.0
Female	74	37.0
Respondents Residence * (*n* = 193)		
Africa	4	2.1
The Americas	21	10.9
South-East Asia	14	7.3
Europe	127	65.8
Eastern Mediterranean	8	4.1
Western Pacific	19	9.8
**Educational Status (*n* = 195)**		
Secondary or lower	9	4.6
High School	57	29.2
University	129	66.2
**Marital Status (*n* = 197)**		
Single/In Relationship	77	39.1
Married	108	54.8
Divorced/Widow(er)	12	6.1
**Smoking Status (*n* = 200)**		
Current Smoker	100	50.0
Ex-smoker	20	10.0
Non-Smoker	80	40.0
**Number of visit to Thailand (*n* = 193)**		
First time	87	45.1
2–3 Times	50	25.9
More than 3 times	56	29.0
Respondents Views on coming to Thailand during 100% SMF ** (*n* = 199)		
Yes	131	65.8
Might	21	10.6
Not Sure	23	11.6
No	24	12.0

* 43 countries respondents taken in the study classified into WHO six regions and ** SMF = Smoke-free Environment.

#### 3.1.2. Perception of the Respondents on Airport Smoke-Free Policy

For measuring the perception, all eight items from questionnaire were computed as a whole. The eight items were as follows: 1. Smoking inside the airport affects the health of both the passengers and workers at the airport. 2. Airports should have a 100% smoke-free environment. 3. A smoking ban would be unfair to smokers. 4. Smoking in the ventilated room does not eliminate the hazards of second hand smoking in the airport. 5. A smoke-free policy is difficult to enforce. 6. Majority of population support smoke-free airport policy. 7. Smoke-free policy is a better method to help smokers to quit smoking. 8. I support smoke-free airports. The 5 levels of perception was further reduced to 3 levels in order to categorized into “Poor”, “Average”, “Good”. The findings on perception of the tourists on airport smoke-free policy showed that males had better perception than females in all three categories of “Poor”. “Average”, and “Good”, as shown in Table 2 below.

**Table 2:** Tourist’s perception levels on airport smoke-free policy by sex.

	Categories of perception (Levels)			Total (%)
	Poor (%)	Average (%)	Good (%)	
Male	19	49	58	126
	(15.1)	(38.9)	(46.0)	(100.0)
Female	16	32	26	74
	(21.6)	(43.2)	(35.1)	(100.0)
	35	81	84	200
	(17.5)	(40.5)	(42.0)	(100.0)

#### 3.1.3. Respondents Support for Smoke-Free Policy

When asked regarding their support for smoke-free policies at the airport in the study, it was found that males support ‘Total Ban’ more than females, while females support “Partial Ban” more than males, as shown in Table 3.

**Table 3:** Support for smoke-free policies among the respondents by sex.

Sex	Choice for smoke-free policy at the airport among the respondents			Total (%)
	No Ban (%)	Partial Ban (%)	Total Ban (%)	
Male	3 (2.4)	58 (46.4)	64 (51.2)	125 (100.0)
Female	0 (0.0)	45 (60.8)	29 (39.2)	74 (100.0)
Total	3 (1.5)	103 (51.8)	93 (46.7)	199 (100.0)

### 3.2. Findings from Inferential Statistics

To explore the associations and relationships among the dependent and independent variables, Chi-square test, Fisher Exact test, one way ANOVA analysis and bivariate logistic regression analysis were adopted. Based on these analyses this section describes the association and relationships between the variables.

#### 3.2.1. Association between General Characteristics and Perception on Smoke-Free Policy (Chi-Square Test Statistics)

The number, percentage and Chi-square values obtained from the findings are listed in Table 4 below. The table clearly shows that no statistically significant association existed between the general characteristic variable and level of perception, except for current smoking status of the respondents, at 5% level of significance with p-value less than 0.001 and chi-square value 19.568 at df = 2.

**Table 4:** Association between general characteristics and perception on smoke-free policy.

General Characteristics	Total (*n*)	Levels of Perception (%)			χ ^2^
		Poor Number	Fair Number	Good	
**Age Group (years)**	**200**				
18–35	114	21 (18.4)	51 (44.7)	42 (36.8)	2.983
35+	86	14 (16.3)	30 (34.8)	42 (48.8)	
Sex: Male	126	19 (15.1)	49 (38.8)	58 (46.0)	2.676
Female	74	16 (21.6)	32 (43.2)	26 (35.1)	
**Educational Status**	**195**				
Up to High School	66	14 (21.2)	25 (37.9)	27 (40.9)	2.312
University	129	21 (16.3)	54 (41.9)	54 (41.9)	
**Marital Status**	**198**				
Single/In Relationship	108	22 (20.4)	47 (43.5)	39 (36.1)	7.691
Married	77	9 (11.7)	30 (39.0)	38 (49.4)	
Divorced/Widow(er)	12	4 (33.3)	2 (16.7)	6 (50.0)	
**Current smoking Status**	200				
Non-Smoker	100	28 (28.0)	42 (42.0)	30 (30.0)	19.568 **
Smoker	100	7 (7.0)	39 (39.0)	54 (54.0)	
**No. of visit to Thailand**	**193**				
First time	87	13 (14.9)	40 (46.0)	34 (39.1)	8.982
2–3 Times	50	6 (12.0)	23 (46.0)	21 (42.0)	
More than 3 times	56	15 (26.8)	14 (25.0)	27 (48.2)	
**Knowledge levels**	**200**				
Poor	147	16 (10.9)	57 (38.8)	74 (50.3)	24.152 **
Average	35	12 (34.3)	15 (42.9)	8 (14.7)	
Good	18	7 (38.9)	9 (50.0)	2 (11.1)	

**p < 0.001.

#### 3.2.2. Support for 100% Smoke-Free Policy

In studying factors associating with the smoke-free policy, results from multinomial logistic regression (Table 5) indicate that there are three dependent variables which associate with support for a 100% smoke-free policy, with statistical significance at *p*-value = 0.05. They are tourists’ level of perception, region of respondent residence, and presence of Designated Smoking Room (DSR).

Comparing ‘average’ category group as level of perception, with reference category group of ‘poor’, results indicates that the likelihood for support for a 100% smoke-free policy is 13.324. Comparing the ‘good’ category group as level of perception, with reference to the category group of ‘poor’, the results indicate that likelihood of support for a 100% smoke-free policy is 3.854. Respondents with good perception had a better support for the policy, which is statistically significant. 

Comparing the region of respondent residence with reference region of Africa, the likelihood of support for a 100% smoke-free policy is 8.334. If comparing European region of residence to the reference region of Africa, the likelihood of support for a 100% smoke-free policy is 7.514. If DSR is present at the airport, the likelihood of support for a 100% smoke-free policy is 0.254 (Table A4 in Appendix section).

**Table 5:** Likelihood Ratio Tests.

Effect	Model Fitting Criteria	Likelihood Ratio Tests		
	−2 Log Likelihood of Reduced Model	Chi-Square	df	Sig.
Intercept	137.025 ^a^	0.000	0	
SEX	137.156	0.131	1	0.717
MRTL_STAT	138.323	1.298	1	0.255
Knowledge_level	137.767	0.742	2	0.690
Age_group	138.741	1.715	1	0.190
Level_Perception	152.628	15.603	2	0.000
Country_Group	156.317	19.292	5	0.002
Come_again, Opinion on visiting Thailand	141.849	4.824	2	0.090
Awareness on smoke-free	138.353	1.328	1	0.249
Fine for violating the policy	137.987	0.962	1	0.327
Pressence of DSRs	141.218	4.192	1	0.041

The chi-square statistic is the difference in −2 log-likelihoods between the final model and a reduced model. The reduced model is formed by omitting an effect from the final model. The null hypothesis is that all parameters of that effect are 0; ^a^ This reduced model is equivalent to the final model because omitting the effect does not increase the degrees of freedom.

## 4. Discussion

Different studies have been done in restaurants, bars and hotel lobbies. The purpose of this study was to determine the perception and support of the tourists regarding smoke-free policy at Suvarnabhumi International Airport, Bangkok, Thailand. In this study the findings showed that current smoking status, knowledge of airport smoke-free policy, respondents residence (WHO regions) were significantly associated with their perception of the smoke-free policy at the airport. Also, existing knowledge, awareness statements about airport smoking and smoking places, fine for flouting the law and presence of DSRs were significantly associated with their support for a 100% smoke-free policy. It was also found that smokers had better knowledge and better perception of smoke-free policy at the airport than non-smokers. Respondents from the Eastern Mediterranean and South-East Asia regions, with poor perception of the policy, had a better support for the 100% smoke-free policy.

This was a pilot study, which had weakness in that the sample was non-randomized, but rather was an incidental sample. This study was conducted among a transient tourist population, which depended on seasonal and timing of traveling. Therefore, it was not possible to control for bias. However, researchers were trying to control bias by exploring data distribution and normality.

The findings from this study may be used to better understanding perceptions on the policy which can either encourage or discourage smoke-free policy and it is critical to help inform and consolidate tobacco control policy. It could help policy makers and implementers to plan and design programmes towards enhancing awareness through education, advocacy and better enforcement of the policy. This results and recommendations will provide the outline of changes that needs to be brought about the existing policy at the airport and ultimately can contribute in initiating the foundation towards effective comprehensive policy at the airport.

### 4.1. General Characteristics

Most respondents surveyed were middle aged adults (aged 18–35 years) and more than half of the respondents were male (sex ratio: 0.587). The findings were consistent with the study done among tourist in air conditioned hotel lobbies in Thailand [19] and Turkey [20].

The tourists taken in the study were mainly European (65.8%); these findings were similar to the study among tourists in Thailand as well but in contrast to the residence region where they were mainly Asians (41%) and white people (38%) Exactly half (50%) of the respondents taken in the study were smokers, 40% non-smokers and 10% ex-smokers and were similar with one of the studies among tourists in Thailand in terms of ex-smokers and smokers but non-smokers were in majority (47.9%). Also the findings were consistent with the study done in Turkey among workers.

The new information from the study is the number of visit made to Thailand, it was found that 45.1% of the respondents were on their first visit and 29% had already visited more than three times. Moreover, 65.8% indicated that they would visit Thailand again if a 100% smoke-free regulation were in place, 23.2% were unsure and only 12.1% indicated that they would not visit Thailand. The findings were similar to the one done among tourists in Thailand.

### 4.2. Awareness about the Smoke-Free Policy at the Airport

The findings showed that only 26.5% of the respondents were aware of the smoke-free policy at the airport, *i.e.*, 45% of smokers and 8% of non-smokers were aware of the smoke-free policy. Among 200 respondents 35% knew about smoking being allowed at the airport, 25.6% knew about the presence of DSRs at the airport, 27.4% knew about the fine for flouting the law. These findings were consistent with a study done in Morocco. When compared to the study done in Turkey, the findings from this study were very poor in terms of awareness [21].

### 4.3. Perception of the Smoke-Free Policy

The findings from the study had shown that 42% of the respondents had a good and 40.5% had a fair perception of the smoke-free policy at the airport. It was also found that current smoking status (*p* ≤ 0.001), awareness levels (*p* ≤ 0.001) and residence of the respondents (*p* ≤ 0.001) were related in a statistically significantly manner to the perception of the smoke-free policy. None of the previous studies had explored the perception. None of the studies among the tourists at the airport had been found to measure the perception of the smoke-free policy.

### 4.4. Support for 100% Smoke-Free Policy

The findings revealed that a majority (partial ban 51.8% and total ban 46.7%) of the respondents supported a partial ban policy. The majority (58%) of current smokers supported a 100% smoke-free policy. Similar results were found in a study of 19 states in America [22] and the UK [23]. But a study from Ghana contradicts the show of strong support (97%) for comprehensive smoke-free legislation, particularly among Christians and Muslims [24]. Studies have revealed that once the comprehensive policy is implemented, within short time the support rises up significantly. From the study it was found that all the tourists from Argentina, Bangladesh, China, Estonia, Ireland, Malaysia, Maldives, Myanmar, New Zealand, Papua New Guinea, UAE and Vietnam had totally supported the smoke-free policy whereas the majority of the tourists from Australia, Denmark, England, Nepal and Sweden had supported it, butonly 50% of the tourists from USA, Switzerland, Bahrain, Brazil, Israel and Netherland and among the tourists from rest of the countries (out of 43 countries) either a minority or no support at all for the smoke-free policy was found. Results reflected that awareness and perception of the smoke-free policy are significantly associated and the respondent’s residence as well as perception had a predictive relationship with the support for a smoke-free policy.

## 5. Conclusions

Based on the responses obtained from 200 departing tourists on the 4th floor of Suvarnabhumi International Airport, a smoke-free policy is well supported and needs strict enforcement so as to improve awareness among them and enhance the perception towards the smoke-free policy at the airport. It was found that around 75% of respondents were unaware of the smoke-free policy at the airport and two-third of respondents would like to come to Thailand even when it is 100% smoke-free. In the study association of general characteristics such as age, gender, educational status, marital status, smoking status, number of visit to Thailand, residence of the respondents, awareness on airport smoke-free policy, levels of knowledge with perception and support for 100% smoke-free policy were explored.

The results showed that two factors, current smoking status, and knowledge levels had a statistically significant association with perception. Likewise, three factors had significant association with the support for the 100% smoke-free policy. They are tourists’ level of perception, region of respondent residence, and presence of Designated Smoking Rooms (DSRs). All other factors taken into account did not reveal any association with the perception and support for a smoke-free policy.

## 6. Recommendations

The following recommendations are based on the research findings and aimed at organizations and other concerned authorities.

### 6.1. At the Institutional Level

1. Since the study findings revealed that almost three fourths of the respondents were unaware of the airport smoke-free policy, the airport authority needs an effective display of the no smoking warning.
2. It has also been found that some of the respondents smoke in the toilet and rest rooms which is against the policy, so to minimize risk from such acts, airport authorities at least can have information on the presence of Designated Smoking Rooms inside the airport.
3. Haphazard smoking allowed outside the building at the airport keeps at stake the health of all, so it needs proper management and an effective monitoring system is the need at the airport in order to move towards a 100% smoke-free environment.

### 6.2. Policy Level

4. Almost all of the respondents were of the view that airport should be smoke-free; this needs to be taken into account by policy makers.
5. For effectively communicating the smoke-free policy among the public regardless of the nationality to raise the awareness and ultimately protect the people, there is a need for a strong education, advocacy and enforcement programme with a monitoring system in place.
6. Perception of the smoke-free policy can help in explaining the support for the law, so there is need for further research in the area among both patrons and the workers at the airport which could clearly come up the support for a 100% smoke-free airport. This would help in establishing an effective comprehensive policy in the airport and ultimately ensure the right to breathe toxin-free air.

## Appendix

**Table A1:** Case Processing Summary.

Variables		*n*	Marginal Percentage
Support 100% smoke free policy	Yes	86	46.0%
	No	101	54.0%
Categories of knowledge (level)	Poor	143	76.5%
	Average	26	13.9%
	Good	18	9.6%
Age grouped into two categories	18–35 YEARS	107	57.2%
	Above 35 years	80	42.8%
Categories of perception (Levels)	poor	33	17.6%
	Average	75	40.1%
	Good	79	42.2%
Grouped countries according to WHO	Africa	4	2.1%
	The Americas	20	10.7%
	South-East Asia	13	7.0%
	Europe	123	65.8%
	Eastern Mediterranean	8	4.3%
	Western Pacific	19	10.2%
Come_again	Yes	125	66.8%
	might	40	21.4%
	No	22	11.8%
Awareness	Yes	64	34.2%
	no	123	65.8%
Fine for violating	yes	64	34.2%
	no	123	65.8%
DSRs presence	yes	48	25.7%
	no	139	74.3%
Valid		187	100.0%
Missing		13	
Total		200	
Subpopulation		142 ^a^	

^a^ The dependent variable has only one value observed in 128 (90.1%) subpopulations.

**Table A2:** Model Fitting Information.

Model	Model Fitting Criteria	Likelihood Ratio Tests		
	−2 Log Likelihood	Chi-Square	df	Sig.
Intercept Only	228.270			
Final	137.025	91.245	17	0.000

**Table A3:** Pseudo R-Square.

Cox and Snell	0.386
Nagelkerke	0.516
McFadden	0.354

**Table A4:** Parameter Estimates.

Support 100% smoke free policy ^a^	B	Std. Error	Wald	df	Sig.	Exp(B)	95% Confidence Interval for Exp(B)	
Intercept							Lower Bound	Upper Bound
SEX	−0.162	0.449	0.131	1	0.718	0.850		2.051
MRTL_STAT	0.422	0.370	1.303	1	0.254	1.526	0.739	3.151
[Knowledge_level = 1]	−1.065	1.524	0.488	1	0.485	0.345	0.017	6.839
[Knowledge_level = 2]	−1.111	1.337	0.690	1	0.406	0.329	0.024	4.524
[Knowledge_level = 3]	0 ^b^			0				
[Age_group = 1]	0.546	0.418	1.704	1	0.192	1.726	0.761	3.914
[Age_group = 2]	0 ^b^			0				
[Level_Perception = 1]	2.590	0.849	9.298	1	0.002	13.324	2.522	70.389
[Level_Perception = 2]	1.349	0.446	9.153	1	0.002	3.854	1.608	9.234
[Level_Perception = 3]	0^ b^			0				
[Country_Group = 1]	2.224	1.474	2.276	1	0.131	9.247	0.514	166.315
[Country_Group = 2]	2.120	0.902	5.530	1	0.019	8.334	1.424	48.784
[Country_Group = 3]	−0.912	1.181	0.597	1	0.440	0.402	0.040	4.064
[Country_Group = 4]	2.017	0.760	7.040	1	0.008	7.514	1.694	33.329
[Country_Group = 5]	0.774	1.199	0.417	1	0.518	2.169	0.207	22.744
[Country_Group = 6]	0^ b^			0				
[co_again = 1.00]	−1.402	0.915	2.349	1	0.125	0.246	0.041	1.478
[co_again = 2.00]	−0.440	0.972	0.205	1	0.650	0.644	0.096	4.323
[co_again = 3.00]	0 ^b^			0				
[aware = 1.00]	−0.751	0.659	1.297	1	0.255	0.472	0.130	1.718
[aware = 2.00]	0 ^b^			0				
[FineV = 1.00]	0.482	0.493	0.954	1	0.329	1.619	0.616	4.254
[FineV = 2.00]	0 ^b^			0				
[DSR_s = 0]	−1.369	0.707	3.753	1	0.043	0.254	0.064	1.016
[DSR_s = 1]	0 ^b^			0				

^a^ The reference category is: Yes; ^b^ This parameter is set to zero because it is redundant.
